# Evaluating the shear bond strength of enamel and dentin with or without etching: A comparative study between dimethacrylate-based and silorane-based adhesives

**DOI:** 10.4317/jced.52322

**Published:** 2015-12-01

**Authors:** Hila Hajizadeh, Atefeh Nemati-Karimooy, Atefeh Nasseh, Naim Rahmanpour

**Affiliations:** 1 DDS, MS, Associated Professor of Restorative Dentistry, Dental Research Center, School of Dentistry, Mashhad University of Medical Sciences, Mashhad, Iran; 2 DDS, Postgraduate Student, Department of Restorative Dentistry, School of Dentistry, Mashhad University of Medical Sciences, Mashhad, Iran; 3 Oral and Maxillofacial Pathologist, Private Researcher, Mashhad, Iran; 4 DDS, Private Practice, Mashhad, Iran

## Abstract

**Background:**

Silorane-based composites and their specific self-etch adhesive were introduced to conquest the polymerization shrinkage of methacrylate-based composites. It has been shown that additional etching of enamel and dentin can improve the bond strength of self-etch methacrylate-based adhesives but this claim is not apparent about silorane-based adhesives. Our objective was to compare the shear bond strength (SBS) of enamel and dentin between silorane-based adhesive resin and a methacrylate-based resin with or without additional etching.

**Material and Methods:**

40 sound human premolars were prepared and divided into two groups: 1- Filtek P60 composite and Clearfil SE Bond adhesive; 2- Filtek P90 composite and Silorane adhesive. Each group divided into two subgroups: with or without additional etching. For additional etching, 37% acid phosphoric was applied before bonding procedure. A cylinder of the composite was bonded to the surface. After 24 hours storage and 500 thermo cycling between 5-55°C, shear bond strength was assessed with the cross head speed of 0.5 mm/min. Then, bonded surfaces were observed under stereomicroscope to determine the failure mode. Data were analyzed with two-way ANOVA and Fischer exact test.

**Results:**

Shear bond strength of Filtek P60 composite was significantly higher than Filtek P90 composite both in enamel and dentin surfaces (*P*<0.05). However, additional etching had no significant effect on shear bond strength in enamel or dentin for each of the composites (*P*>0.05). There was no interaction between composite type and additional etching (*P*>0.05). Failure pattern was mainly adhesive and no significant correlation was found between failure and composite type or additional etching (*P*>0.05).

**Conclusions:**

Shear bond strength of methacrylate-based composite was significantly higher than silorane-based composite both in enamel and dentin surfaces and additional etching had no significant effect on shear bond strength in enamel or dentin for each of the composites. The mode of failure had no meaningful relation to the type of composite and etching factor.

** Key words:**Shear bond strength, adhesive, composite resin, silorane, methacrylate.

## Introduction

Since 1954 that Bonocore introduced acid etching procedure as a pretreatment method that enhances the strength bonding of composite resins to enamel for the first time (1), and then it’s clinical application presented in 1976 by Cueto and Bonocore (2), many trials have been done to improve the quality of composites and adhesives and several pretreatment methods were discovered and presented.

In fact, adhesive generations from first to seventh, are the results of these studies that became more modified and their application became easier step by step, so that along with sixth generation of adhesives, the term self-etch came into the world of restorative dentistry (3). Self-etching is based on the use of acidic resin monomers that allow simultaneous demineralization and infiltration of the partially demineralized substrate by resin monomers (4).

The composite resins that are mainly used, which are methacrylate-based, have some imperfections such as shrinkage after polymerization. As these composites are a mixture of different methacrylate monomers that polymerize linearly, their shrinkage can negatively compromise the longevity of resin-based restorations, resulting in unsatisfactory marginal adaptation, marginal discoloration, decrease in surface texture, secondary caries and excessive loss of anatomic form (5-9).

With the purpose of overcome this imperfection, scientists introduced silorane-based resins that have ring-shaped monomers and have low polymerization shrinkage and hydrophobicity property.

In recent years, many studies have done about structural and clinical benefits of silorane composites.

But among these researches, a few has pointed to the effect of additional etching of dental substrate before applying silorane-based adhesives on shear bond strength (SBS) of dentin and enamel.

Some previous trials revealed that the shear bonding strength of the enamel and dentin enhances by etching dental substrates with (35%) phosphoric acid (10-14), but some studies indicated that pretreatment of enamel with phosphoric acid before application of two-step self-etch adhesives does not improve bond strength or even can decrease it (8,9). Furthermore, when we consider dentin as a dental substrate that unintentionally exposed to acid while we attempt to etch only enamel, and with the concern that dentin bond strength may be adversely affected by etching (8,12,14,15), the necessity of survey about strength bonding of dentin after etching becomes obvious.

Considering that there are a few papers about the effect of phosphoric acid etching before the application of silorane adhesive on the shear bond strength of dentin and enamel (16,17), we accomplished this study and compared the shear bond strength of silorane-based adhesive of enamel and dentin with a well-known methacrylate-based two-step self-etch adhesive system such as Clearfil SE Bond adhesive with and without an additional phosphoric acid etch.

## Material and Methods

Present study was an *in vitro* interventional parallel study with these purposes: comparing the shear bond strength of silorane-based adhesive to enamel and dentin and a well-known methacrylate-based adhesive (Clearfil SE Bond) with or without additional etching and also, evaluation of the failure mode.

According to data in Giacobbi and Vandewalle paper (18) and PASS test in NCSS software with α=0.05, sample size has detected as n=10 for each group. We collected 40 extracted human sound premolars which were extracted because of therapeutic reasons (i.e. orthodontic treatments) and Ethical Committee of Mashhad University of Medical Sciences confirmed our sample selection.

Inclusion criteria in this study were: (1) human premolar tooth, (2) tooth must be completely sound. Exclusion criteria were: (1) teeth with any fracture, decay, filling or attrition, (2) first mandibular premolar (because of insufficient amount of dental mass), (3) storage in a dry environment after extraction.

**Figure 1:**
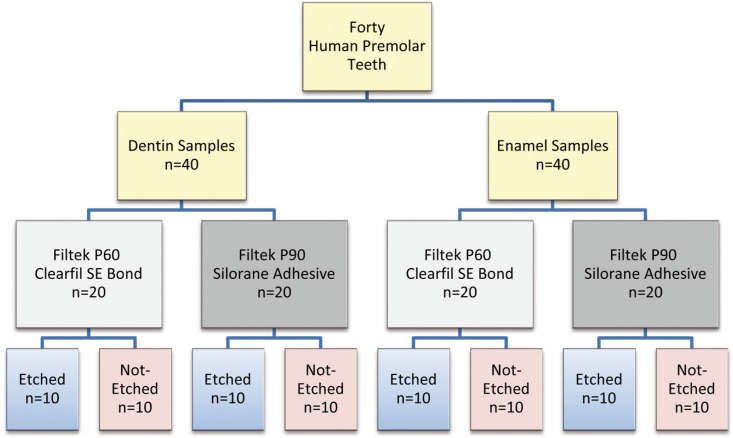
Classifying samples based on dental substrate (enamel and dentin), adhesive system (Filtek P60 with Clearfil SE Bond and Filtek P90 with Silorane Adhesive) and the type of pretreatment (etched and not-etched).

After debridement, the teeth were stored in 0.5 % chloramine solution for one week and then were transferred into distilled water until the beginning of the experimental phase. Each tooth sectioned three times perpendicular to the line axis of the tooth; with a low-speed diamond saw (IsoMet, Buehler, USA) under a water coolant. The first cut created a smooth enamel surface. The second cut was about 2 mm under dentin-enamel junction and the third one was 2 mm more apically to the second cut. So, two dental specimens, each with about 2 mm thickness were obtained that one had a smooth enamel surface and the other had a smooth dentin surface. Thus, we had 80 dental samples; 40 enamel samples and 40 dentin samples. Dentin or enamel samples randomly divided into subgroups, according to composite type with its adhesive and applying additional etching. So, eight experimental groups were obtained. Classification of the groups is shown in figure 1.

We used two composite resins with their compatible bonding systems. Half of the samples received a methacrylate-based composite resin (Filtek P60, 3M, USA) with a two-step self-etch adhesive (Clearfil SE Bond, Kurary, Japan), while the other half received a silorane-based composite resin (Filtek P90, 3M, USA) and its specific adhesive (Silorane System Adhesive, 3M, USA).

**Figure 2:**
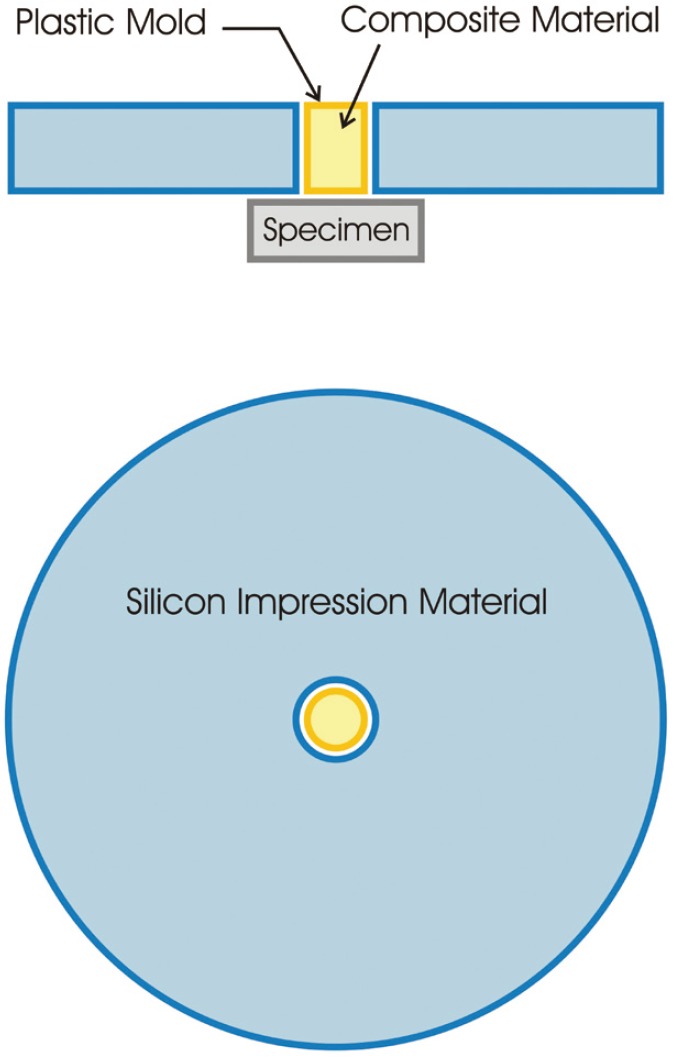
Schematic figure of composite cylinder construction on specimen.

**Figure 3:**
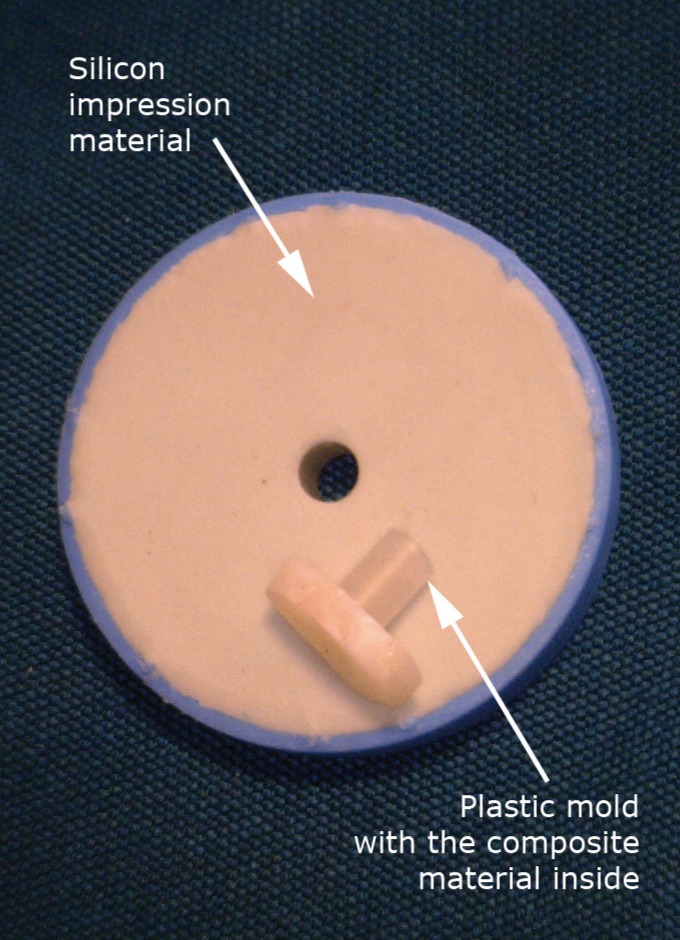
Prepared model of composite cylinder on specimen by plastic mold and silicon impression material.

The adhesives and composites were applied according to the manufacturer instructions (without etching), or they were applied with additional etching. The etched groups were treated with (35%) phosphoric acid (Ultra-etch, Ultradent, USA) for 15 seconds, rinsed and air-dried gently before the application of the adhesives. An LED light curing unit (Bluephase, Ivoclar-Vivadent, Liechtenstein), with a power density of 600 mW/cm2 for 20 seconds was used to cure the adhesive. Immediately after polymerization of the adhesive, a cylindrical plastic mold (2.5 mm in diameter and 3.0 mm in height), was fixed on the prepared surface of specimen by silicon impression material, which then filled with two layers of composite resin (according to the study groups), each with the thickness of 1.5 mm (Figs. 2,3) and polymerized for 40 seconds with a power density of 600 mW/cm2. The mold was cut with a scalpel. In order to simulate oral cavity circumstances, the specimens were stored in water for 24 hours, and then were thermo-cycled for 500 cycles at 5-55°C for 60 seconds. The interval time between each thermo-cycling phase was 30 seconds. After 7 days of storage in water, the bottom surface of each specimen was attached to the pulling device using cyanoacrylate glue (Alpha Glue, Razi Chemical Co., Iran). The shear bond strength was evaluated with a universal testing machine (STM-20, Santam, Iran) using a 0.5 mm crosshead blade, loaded at a crosshead speed of 0.5 mm/min. A notched crosshead matched with the diameter of the bonded cylinder was used to apply the load. The load required to de-bond the specimen was recorded and expressed in MPa and the descriptive statistics were determined.

To check the normality of data distribution, Kolmogorov-Smirnov Test was used. Two separate two-way analysis of variance tests (ANOVA) were conducted for shear bond strength of enamel and dentin substrates with the main variables adhesive type (Clearfil SE Bond vs. Silorane Adhesive ) and conditioning technique (etched vs. not-etched). The post-hoc multiple comparisons Tukey’s test was used for pair wise comparisons between the groups. Significance was determined at a probability of *p*<0.05. All statistical analysis was performed by Statistical Package for Social Sciences (SPSS) version 11.5 (SPSS Inc, USA).

The observation and analysis of the failure type was conducted by one trained evaluator, with a stereomicroscope at 20x magnification. The fractures in interfaces were classified as adhesive, cohesive in enamel or dentin or composite, or mixed. The data of failure mode was assessed using Chi square analysis and Fissure exact test.

## Results

For both enamel and dentin, the interactions between the two variables adhesive type and conditioning technique were shown not to be significant. The mean shear bond strength for the two adhesives evaluated to enamel and dentin substrates are summarized in  Table 1. Although Clearfil SE Bond demonstrated increased bond strengths to enamel and dentin when applied followed treatment with phosphoric acid, the differences were not significant (*p*>0.05). The decrement in mean bond strengths to enamel and dentin for Silorane System Adhesive, following phosphoric acid etching, was not significant too (*p*>0.05). Only adhesive type demonstrated a significant effect on shear bond strength to enamel and dentin (*p*<0.05). For both enamel and dentin, whether the adhesives were applied following phosphoric acid treatment or according to the manufacturer’s instructions without phosphoric acid etching, the mean bond strength values were significantly higher for Clearfil SE Bond than Silorane System Adhesive (*p*<0.05). Clearfil SE Bond applied to etched enamel demonstrated the highest mean shear bond strength (91.29 MPa) and Silorane System Adhesive applied to etched dentin showed the lowest mean shear bond strength (46.66 MPa) among all tested groups. Totally, the mean shear bond strengths to dentin specimens were less than their equivalent enamel groups.

**Table 1:**
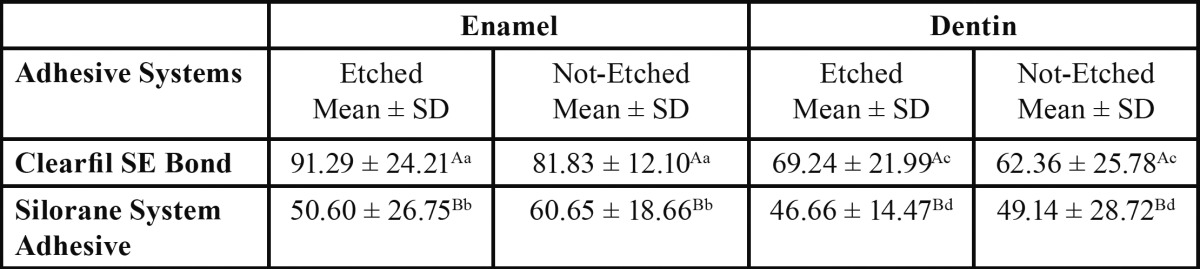
Mean shear bond strength results for both adhesives applied to phosphoric acid etched vs. not-etched dental substrates (n=80).

Failure mode distribution of the specimens bonded to enamel and dentin were also assessed. None of the specimens showed cohesive failures, and adhesive failures were the most prevalent type of failure. Fisher Exact test showed that neither adhesive type nor conditioning technique had any significant effect on failure mode.

## Discussion

While some researchers showed additional etching of enamel and dentin enhances bonding strength of two-step self-etch adhesive and silorane system adhesive (17,19,20), we found no significant difference between shear bond strength of etched and not-etched enamel and dentin. On the other hand, we surprised when we found that the type of adhesive had a noticeable effect on shear bond strength of enamel and dentin. Our results were compatible with data of a previous study that was done by Adebayo *et al.* (5), and showed higher bond strengths of the two-step self-etch adhesive than Silorane System Adhesive. But this finding is opposite with the results of Giacobbi and Vandewalle (18), Koliniotou-Koumpia *et al.* (21), Sampaio *et al.* (22). These authors said there is no difference between Silorane System Adhesive and a methacrylate-based adhesive system. This inconsistency between our results and theirs may be contributed to different self-etch adhesives and variety of composite resins that had been used in researches. Although all composite resins have methacrylate bases but they are different in chemical structure and components. On the other hand our scale for evaluating of bond strength was shear bond strength similar to studies that performed by Ustunkol *et al.* (17), Koliniotou-Koumpia *et al.* (21), and Barta *et al.* (19). This scale is one of the most common criterions for this purpose whereas the test used in Sampaio *et al.* (22) and Giacobbi and Vandewalle (18) researches was micro-tensile bond strength that is sensitive to minute changes in measurements.

Present study revealed that shear bond strength of Silorane System Adhesive is obviously less than shear bond strength of Clearfil SE Bond (*p*<0.05). When we consider that Clearfil SE Bond has 10-MDP monomer that is able to connect to calcium content in enamel and dentin (23), while Silorane System Adhesive has not this monomer, the lower shear bond strength seems logical.

However, inconsistency between our results and previous studies may be influenced by many factors such as failure test, cross-head speed, depth of dental substrate that was examined, aging process, type of storage solution and time of storage after tooth extraction.

In present study, additional etching with phosphoric acid (35%), had no influence on shear bond strength of enamel and dentin (*p*>0.05). Although this finding is similar to Pucci *et al.* (16) achievements, but it is contradicted with Ustunkol *et al.* (17), Batra *et al.* (19) and Taschner *et al.* (20) findings that claimed etching process has a significant effect on shear bond strength of Silorane System Adhesive and self-etch adhesives.

Although Manuja *et al.* (24) reported that etching of enamel can decrease microleakage in self-etch adhesives, study that perfor-med by Rengo *et al.* (9) showed etching process increased the microleakage in dentin area.

When we compare Silorane System Adhesive and Clearfil SE Bond, Silorane System Adhesive has a higher viscosity. Thus, can-not be fluent as Clearfil SE Bond on the etched enamel and dentin. As we make porosities in enamel and dentin by acid-etching, Silorane System Adhesive didn’t emerge into these porosities as well as Clearfil SE Bond. So, shear bond strength of Clearfil SE Bond is higher than Silorane System Adhesive.

As stated previously, etching of dentin can decrease bonding quality of self-etch adhesives (9). But while we are conditioning a dental cavity including both enamel and dentin, we are not sure that dentin is protected from acid. Thus, we examined dentin as a dental substrate for applying additional etching to evaluate that if etching process decreases shear bond strength to dentin. The present study, confirmed the previous findings, so, more temptation must be applied for protecting dentin from etching process.

In this trial, we attempt to simulate oral cavity environment. Although what we accomplished, doesn’t exactly match the clinical conditions, the study results in this situation are closer to the reality and making decisions based on these consequences will be more accurate.

And our Final achievement in this study: The failure pattern in enamel and dentin is mainly adhesive type that means this fracture happens in bonding zone of composite restoration. This result is similar to other previous studies and was expectable.

## Conclusions

Within the limitations of this study conducted and the results obtained, it can be concluded that additional etching has no significant effect on shear bond strength of enamel and dentin. Also, it was found that shear bond strength of silorane-based material is significantly less than methacrylate-based material. Besides, the mode of failure had no meaningful relation to the type of composite resin and additional etching as a kind of surface treatment.
